# Draft Genome Sequence of the Phosphate-Solubilizing Rhizobacterium Burkholderia pseudomultivorans Strain MPSB1, Isolated from a Copper Mined-Out Site

**DOI:** 10.1128/MRA.01304-20

**Published:** 2021-01-07

**Authors:** Berna Lou L. Aba-Regis, Kristel Mae P. Oliveros, Cherry Ibarra-Romero, Asuncion K. Raymundo, Nelly S. Aggangan, Teofila O. Zulaybar, Albert Remus R. Rosana

**Affiliations:** a Microbiology Division, Institute of Biological Sciences, University of the Philippines Los Baños, College, Laguna, Philippines; b Department of Biological Sciences, Visayas State University, Baybay City, Leyte, Philippines; c Department of Chemistry, University of Alberta, Edmonton, Alberta, Canada; d National Academy of Science and Technology Philippines, Bicutan, Taguig City, Philippines; e National Institute of Molecular Biology and Biotechnology, University of the Philippines Los Baños, College, Laguna, Philippines; Portland State University

## Abstract

Burkholderia pseudomultivorans MPSB1 was isolated from a copper mined-out soil sample collected from Mogpog, Marinduque, Philippines. Here, we report the draft genome sequence with predicted gene inventories supporting rhizosphere bioremediation, such as heavy metal tolerance, phosphate solubilization, and siderophore production.

## ANNOUNCEMENT

Plant growth-promoting rhizobacteria were isolated and characterized from bioremediated and heavy metal-contaminated environments such as mine sites in Philippines (1–3). Members of the *Burkholderia* species that promote plant growth activities and are normally isolated not from infected patients but from environmental sources have increased remarkably (4). Here, we report the draft genome sequence of Burkholderia pseudomultivorans strain MPSB1, isolated from a phytoremediated copper mined-out site in Mogpog, Marinduque, Philippines. The strain was sequenced to provide insights to its potential utilization as a bioremediating inoculum in heavy metal postmining sites in Philippines. A soil sample was taken from a 20-cm depth 10 cm from *Pterocarpus indicus*, the plant used for bioremediation (5).

MPSB1 was isolated by spreading 0.1 ml of 10-fold serial dilutions on National Botanical Research Institutes phosphate (NBRIP) medium containing insoluble tricalcium phosphate (6). The plates were incubated at 37°C for 14 days. Colonies showing halo zones were purified by extensive subculturing on NBRIP medium (7), generating axenic isolate MPSB1. The purity of the isolate was assessed by routine Gram staining, 16S rRNA gene sequencing, and electron microscopy analyses (transmission electron microscopy [TEM] and scanning electron microscopy [SEM]). Genomic DNA was purified from a 48-h culture grown in tryptic soy broth (37°C with shaking at 200 rpm) using the ZymoResearch Quick-DNA fungal/bacterial kit and quantified using NanoDrop and Qubit v2.0. Nextera XT DNA libraries were created and sequenced using the NextSeq reagent kit (2 × 250 bp) (Illumina, San Diego, CA). FastQC v0.11.8 was used to inspect the quality of the sequences, and quality trimming was based on Phred quality scoring 20 and SolexaQA v3.0 (8). Trimmed reads were *de novo* assembled using IDBA-UD v1.1.1 (9) implemented in the Microbial Genome Atlas (MiGA) pipeline v0.3.6.2 (10). Genome completeness was assessed using BUSCO v4.1.4 (11). The draft genome sequence was annotated using the NCBI PGAP v4.8 (12). Taxonomic classification was established using the Type (Strain) Genome v0.90 (13) and Microbial Genome Atlas servers, calculating for the digital DNA:DNA hybridization (dDDH) (14) and average nucleotide identity (ANI) (15), respectively. Secondary bioactive metabolites were predicted using antiSMASH v5.0 (16). Default parameters were used for all software unless otherwise specified.

Paired-end sequencing yielded 1,097,695 reads at 165× coverage. The draft genome represented in 145 contigs (*N*_50_, 88,373 bp) has a G+C content of 67.38% and an estimated size of 7,742,780 bp. The genome of MPSB1 showed an ANI of 95.45% with the closest type strain, Burkholderia pseudomultivorans DSM 105103^T^.

Genome annotation detected 7,021 coding sequences, 2 rRNA genes, and 59 tRNAs. The genome contains predicted genes supporting rhizosphere-associated processes, including metal scavenging by siderophores, quorum sensing by homoserine lactone, heavy metal efflux pumps, and phosphate solubilization. Table 1 highlights 6 of the 14 predicted biosynthetic gene clusters. The genome also contains predicted biosynthetic gene clusters for the production of bacteriocin (17), biopolymers (polyhydroxyalkanoate and exopolysaccharides) (17, 18), and several terpenoids.

**TABLE 1:** Summary of antiSMASH results for Burkholderia pseudomultivorans MPSB1

Predicted biosynthetic metabolite	Contig no.	Coordinates within the contig (nucleotide position)	Similarity to known BGC^*a*^ (%)
Siderophore	14	16571–80539	100 (ornibactin BGC)
Homoserine lactone	15	65458–86066	100 (homoserine lactone BGC)
Bacteriocin	24	6191–17003	50 (linocin BGC)
Terpene	70	19499–39037	50 (*N*-acyloxyacyl glutamine BGC)
Arylpolyene	87	1770–25741	50 (polyhydroxyalkanoate BGC)
Phosphonate	103	1–19037	15 (dehydroformidomycin BGC)

a BGC, biosynthetic gene cluster.

### Data availability.

The whole-genome project for Burkholderia pseudomultivorans MPSB1 has been deposited in DDBJ/ENA/GenBank under accession number JADKRM000000000. The version described in this paper is the first version (JADKRM010000000), under BioProject number PRJNA674354, BioSample number SAMN16631211, and Sequence Read Archive (SRA) number SRR13060793.
